# Evaluation of the Antimicrobial Potential and Toxicity of a Newly Synthesised 4-(4-(Benzylamino)butoxy)-9*H*-carbazole

**DOI:** 10.3390/ijms222312796

**Published:** 2021-11-26

**Authors:** Katarzyna Zawadzka, Aleksandra Felczak, Iwona E. Głowacka, Dorota G. Piotrowska, Katarzyna Lisowska

**Affiliations:** 1 Department of Industrial Microbiology and Biotechnology, Faculty of Biology and Environmental Protection, University of Lodz, 90-237 Lodz, Poland; aleksandra.felczak@biol.uni.lodz.pl (A.F.); katarzyna.lisowska@bioil.uni.lodz.pl (K.L.); 2 Bioorganic Chemistry Laboratory, Faculty of Pharmacy, Medical University of Lodz, 90-151 Lodz, Poland; iwona.glowacka@umed.lodz.pl (I.E.G.); dorota.piotrowska@umed.lodz.pl (D.G.P.)

**Keywords:** antimicrobial activity, carbazole derivative, antibacterial agent, antifungal properties, Gram-positive bacteria, Gram-negative bacteria

## Abstract

One of the greatest threats to human and animal health is posed by infections caused by drug-resistant bacterial strains. Therefore, newly synthesised substances are tested for their antimicrobial activity. Carbazole derivatives seem to be promising antibacterial agents. This study aimed at investigating the toxicity and activity of newly synthesised, functionalised carbazole derivative **2** (4-(4-(benzylamino)butoxy)-9*H*-carbazole) against various microorganisms. Its antimicrobial potential against Gram-positive and Gram-negative bacteria, yeast, and filamentous fungi was examined according to CLSI (Clinical and Laboratory Standards Institute) standards. The tested compound was found to efficiently inhibit the growth of Gram-positive strains. The addition of carbazole derivative **2** at the concentration of 30 µg/mL caused inhibition of bacterial growth by over 95%. Moreover, about 50 and 45% limitation of *Pseudomonas aeruginosa* and *Aspergillus flavus* growth was observed in the samples incubated with the addition of 20 and 60 µg/mL of the compound, respectively. Its addition to the microbial cultures caused an increase in the permeability of the cellular membrane. Slight haemolysis of red blood cells was observed after 24-h treatment with carbazole derivative **2**. On the other hand, human fibroblasts were found to be more sensitive to its effects. The activity of the tested compound indicates a possibility of its further modification in order to obtain effective drugs, especially against drug-resistant staphylococci.

## 1. Introduction

Infectious diseases, especially those caused by drug-resistant microorganisms, are a global problem for clinical and veterinary medicine. Mortality from infections had been very high until the discovery of penicillin by A. Fleming in 1928. Nowadays, microorganisms again pose a serious threat to human health due to the development of resistance mechanisms against antibiotics that have been effective so far [1]. In the United States, multidrug-resistant strains generate health care costs in the amount of USD 4.7 billion and cause about 29000 deaths per year. In Europe, these values are estimated at USD 1.5 billion and 33,000 deaths [2]. According to the report prepared for the UK government, a failure to counteract antimicrobial resistance will result in 10 million deaths globally and the cost at the level of USD 100 trillion [1]. 

The application of conventional antibiotics is one of the most popular strategies for the treatment of microbial infections. However, new methods of combating pathogenic microorganisms are also being developed. These include both searching for new molecules with high antimicrobial properties and using combinations of conventional antibiotics or nanobiotics [2]. 

Carbazole is a tricyclic heteroaromatic compound consisting of two benzene rings fused on either side of a five-membered nitrogen-containing ring. It occurs in coal tar and in plants as carbazole alkaloids possessing different biological properties. A carbazole moiety is a structural unit present in many compounds with pharmacological properties and it is commonly used in the production of dyes or polymers. The biological potency and medical importance of carbazole derivatives are often emphasised in the literature [3]. 

**Figure 1:**
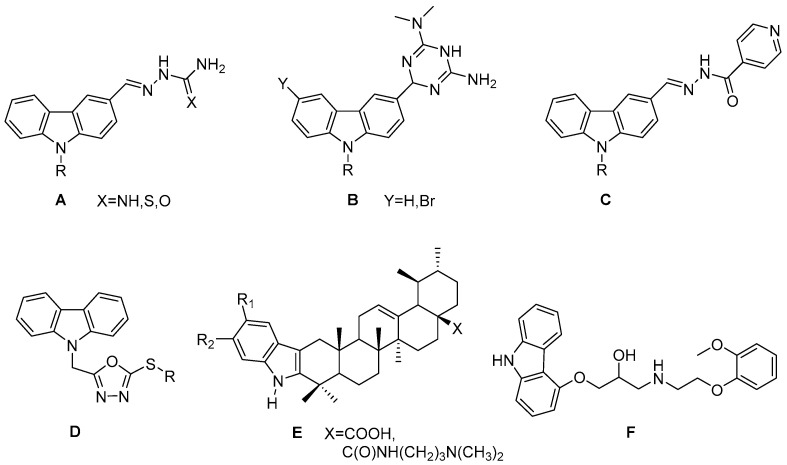
Examples of biologically active carbazole derivatives.

For example, carbazole derivatives **A**–**C** (Figure 1) substituted with aminoguanidine, dihydrotriazine, isonicotinic, semicarbazide, or thiosemicarbazide moiety showed antibacterial and antifungal activities with minimum inhibitory concentrations in the range of 0.5–16 mg/mL [4]. Xie et al. [5] proved the antibacterial potential of carbazole–oxadiazole conjugates **D**, which exhibited higher activity against Gram-positive *Staphylococcus aureus* strains (MIC = 0.6–4.6 nmol/mL) than the reference drug, norfloxacin (MIC = 6–40 nmol/mL). Derivatives **E** having a carbazole unit incorporated in the structure of ursolic acid also occurred to be promising antibacterial agents [6]. Carvedilol **F**, a derivative of carbazole, which is a nonselective ß-adrenergic receptor antagonist also exhibited antimicrobial properties, especially against Gram-positive bacteria (MIC = 40–55 mg/L) [7].

**Scheme 1:**
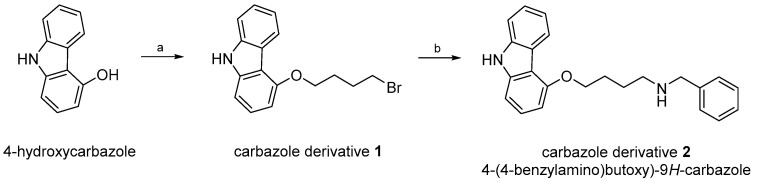
Reaction and conditions: (a) 1,4-dibromobutane, KOH, CH_3_CN, KI, r.t. 24 h [8]; (b) benzylamine, CH_3_CN, KI, reflux, 5 h.

Abundant evidence showing the strong antimicrobial potential of carbazole derivatives prompted us to look for further biologically active analogues. In this paper, the antibacterial and antifungal properties of the newly synthesised, functionalised carbazole derivatives **2** (Scheme 1) are described. Its activity was investigated against aerobic Gram-positive and Gram-negative bacterial strains, as well as against yeast and filamentous fungus cells. Toxicity studies concerning the haemolytic and cytotoxic activity of the compound against mammalian cells were carried out. Its influence on the permeability of microbial membranes was also investigated.

## 2. Results

### 2.1. Synthesis and Characterisation of 4-(4-(Benzylamino)butoxy)-9H-carbazole

Carbazole derivative **2** was synthesised starting from commercially available 4-hydroxycarbazole, which reacted with 1,4-dibromobutane in the presence of potassium hydroxide following the procedure described in the literature and its spectral characteristic was in full agreement with the data reported earlier [8]. The treatment of 4-(4-bromobutoxy)-9*H*-carbazole **1** with benzylamine and potassium iodide in acetonitrile resulted in the formation of carbazole derivative **2** (4-(4-(benzylamino)butoxy)-9*H*-carbazole) in 80% yield [9] (Scheme 1). The structure and purity of the final compound were determined by ^1^H, ^13^C, and IR, as well as by elemental analysis.

### 2.2. Antimicrobial Properties of Carbazole Derivative ***2***

**Figure 2:**
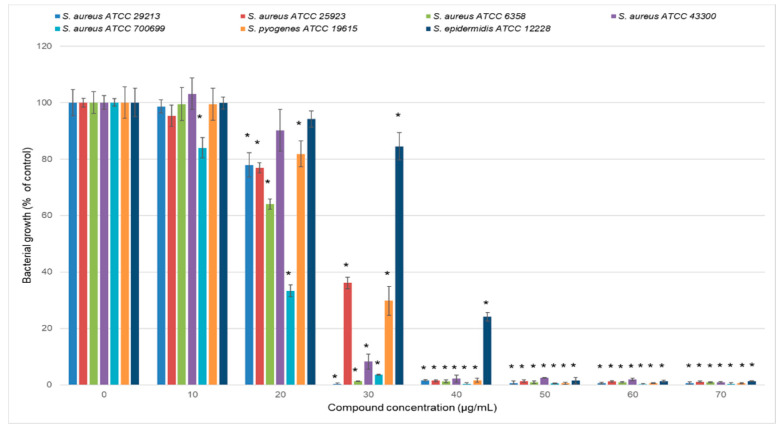
Growth of Gram-positive bacteria in the presence of carbazole derivative **2**. The obtained results were expressed as a percentage of growth of untreated cells in control samples. The data represent mean ± SD of three different experiments performed in triplicate. *P*-values were determined by the one-way analyses of variance (ANOVA) where * represents statistically significant results (*p* ≤ 0.05).

Antimicrobial activity of carbazole derivative **2** was examined against the cells of Gram-positive (*Staphylococcus aureus* ATCC 29213, *Staphylococcus aureus* ATCC 25923, *Staphylococcus aureus* ATCC 6358, *Staphylococcus aureus* ATCC 700699, *Staphylococcus aureus* ATCC 43300, *Staphylococcus epidermidis* ATCC 12228, *Streptococcus pyogenes* ATCC 19615), and Gram-negative (*Escherichia coli* ATCC 25922, *Proteus hauseri* ATCC 13315, *Pseudomonas aeruginosa* ATCC 15442) bacteria, as well as fungi (*Candida albicans* ATCC 10231, *Aspergillus flavus* ATCC 9643). It showed a higher antibacterial potential against Gram-positive bacteria than against Gram-negative strains and fungal cells. Significant inhibition of the growth of most Gram-positive bacteria was observed in the cultures incubated with the addition of the compound at a concentration of 30 µg/mL (Figure 2). Among Gram-positive microorganisms, the lowest value of the minimum inhibitory concentration (MIC) 30 µg/mL was observed in the cultures of *S. aureus* ATCC 29213 and *S. aureus* ATCC 6358 (Table 1). For the remaining staphylococci and the *S. pyogenes* strain, the MIC value was 40 µg/mL. The highest MIC value of 50 µg/mL was found in the Gram-positive *S. epidermidis* cultures. The minimum bactericidal concentrations (MBCs) of carbazole derivative **2** in *S. pyogenes*, *S. epidermidis,* and *S. aureus* ATCC 700699 were 60, 60, and 70 µg/mL, respectively. The values of MBC for the other tested *S. aureus* strains and *S. epidermidis* bacteria were above the range of the tested concentrations of the compound (10–70 µg/mL). 

**Figure 3:**
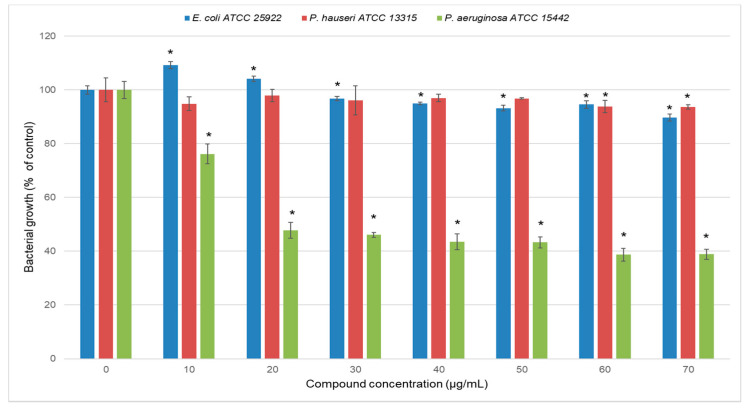
Growth of Gram-negative bacteria in the presence of carbazole derivative **2**. The obtained results were expressed as a percentage of growth of untreated cells in control samples. The data represent mean ± SD of three different experiments performed in triplicate. *P*-values were determined by the one-way analyses of variance (ANOVA) where * represents statistically significant results (*p* ≤ 0.05).

The Gram-negative microorganisms were found to be more resistant to its antimicrobial activity than the Gram-positive strains (Figure 3). A slight reduction in the growth of *E. coli* and *P. hauseri* incubated with the addition of carbazole derivative **2** at the concentrations of 10–70 µg/mL was observed. In the case of *P. aeruginosa*, more than 50% growth inhibition was detected in the cultures treated with 20 µg/mL of the compound. The values of MIC and MBC for the Gram-negative strains were above the range of its tested concentrations (10–70 µg/mL).

**Figure 4:**
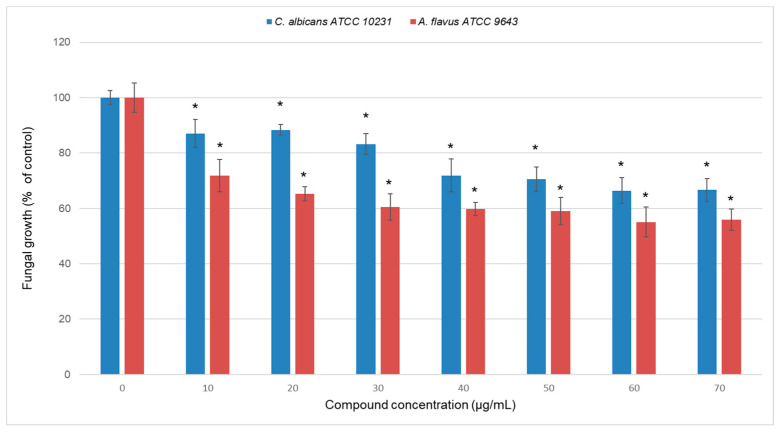
Growth of fungi in the presence of carbazole derivative **2**. The obtained results were expressed as a percentage of growth of untreated cells in control samples. The data represent mean ± SD of three different experiments performed in triplicate. *P*-values were determined by the one-way analyses of variance (ANOVA) where * represents statistically significant results (*p* ≤ 0.05).

The antifungal potential of carbazole derivative **2** was examined against the yeast *C. albicans* ATCC 10231 and the filamentous fungus *A. flavus* ATCC 9643 (Figure 4). The yeast cells were less susceptible to the activity of the compound, compared with the filamentous fungus. An about 35% yeast growth reduction was demonstrated in the *C. albicans* cultures supplemented with the addition of carbazole derivative **2** at the concentration of 50 µg/mL. In the case of *A. flavus*, a 40% inhibition of growth was observed in the samples treated with 50 µg/mL of the compound. The values of MIC and MFC (minimum fungicidal concentration) were above its highest tested concentration (70 µg/mL).

### 2.3. Cytotoxicity and Haemolytic Activity of Carbazole Derivative ***2***

**Figure 5:**
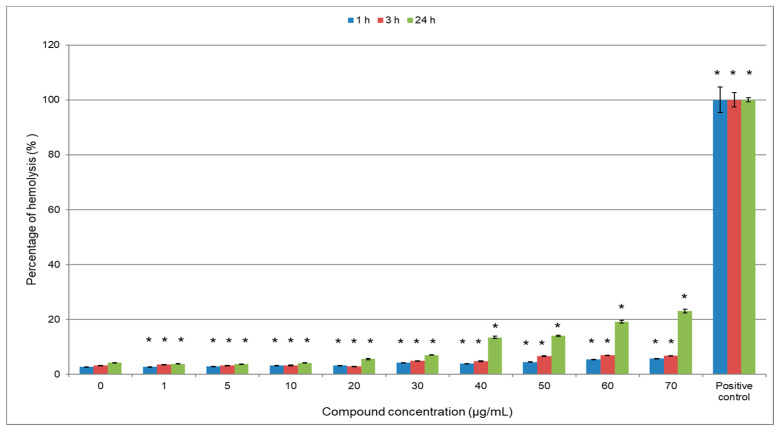
Haemolysis of red blood cells in the presence of carbazole derivative **2**. The obtained results were expressed as a percentage of growth of untreated cells in control samples. The data represent mean ± SD of three different experiments performed in triplicate. *P*-values were determined by the one-way analyses of variance (ANOVA) where * represents statistically significant results (*p* ≤ 0.05).

The haemolytic activity of carbazole derivative **2** was studied in the concentration range of 1–70 µg/mL (Figure 5). The amount of released haemoglobin was measured after 1, 3, and 24 h incubation of the red blood cells with the addition of the compound. The positive control (cells suspended in aseptic deionised water; 100% haemolysis) and negative control (cells incubated without the tested compound) were prepared. The obtained results were presented as a percentage of haemolysis in the positive control samples. The haemolysis process in the samples incubated for 1 and 3 hours with the addition of carbazole derivative **2** at the concentrations 1–70 µg/mL was observed to reach the levels of 2.5–7%. In the case of the erythrocytes treated with the compound for 24 h, about three-fold increase in the haemolytic effect was observed. Its addition at the highest tested concentration induced haemolysis at the level of 23%.

**Figure 6:**
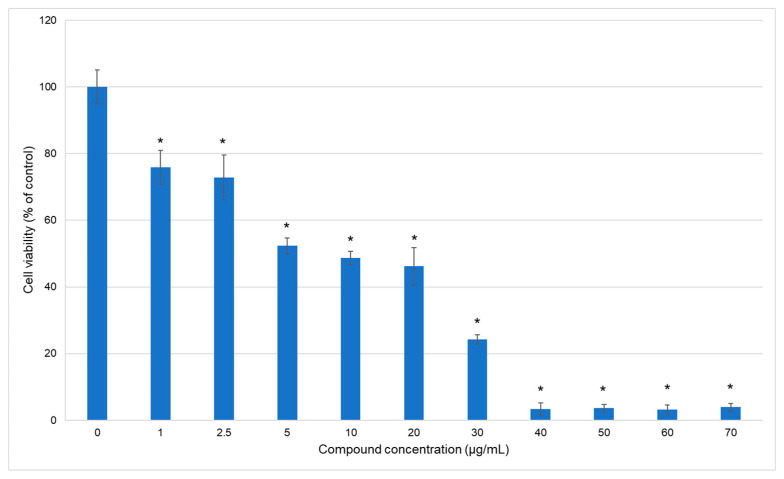
Viability of human fibroblasts treated with carbazole derivative **2**. The obtained results were expressed as a percentage of viability of untreated cells in control samples. The data represent mean ± SD of three different experiments performed in triplicate. *P*-values were determined by the one-way analyses of variance (ANOVA) where * represents statistically significant results (*p* ≤ 0.05).

The cytotoxic activity of carbazole derivative **2** was studied in the concentration range of 1–70 µg/mL (Figure 6). The analysis of the viability of human fibroblasts treated with the compound showed its higher toxicity to fibroblasts than to red blood cells. A decrease in fibroblasts viability by 25, 50, 75, and 97% was observed in the cell cultures supplemented with carbazole derivative **2** at the concentrations 1–2.5, 5–20, 30, and 40–70 µg/mL, respectively. The 24h-IC_50_ was 10 µg/mL.

### 2.4. The Mechanism of Carbazole Derivative ***2*** Antimicrobial Activity

**Figure 7:**
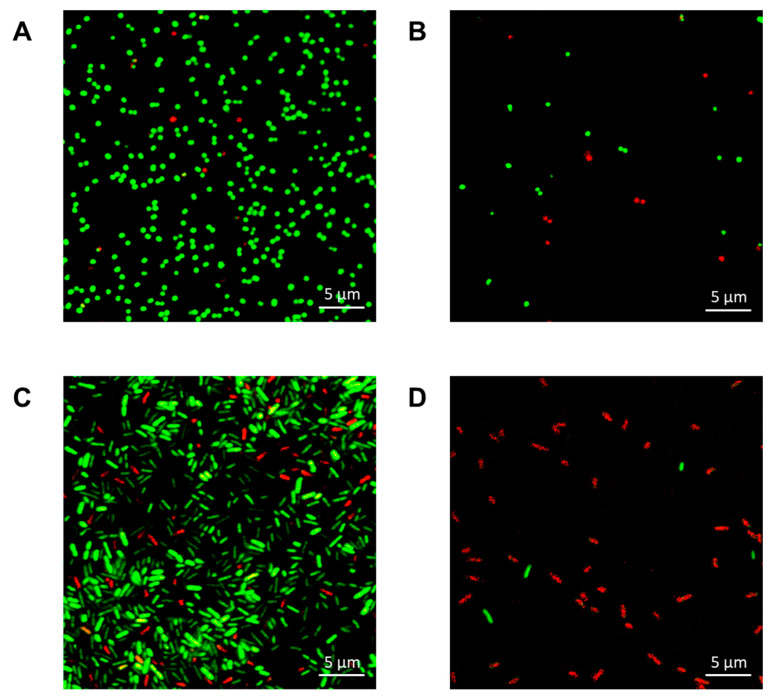
Confocal images of untreated cells of *S. aureus* (**A**) and *P. aeruginosa* (**C**) and bacterial cells treated with carbazole derivative **2** at the concentration of 40 µg/ mL (**B**,**D**).

The analysis of the confocal images of bacteria stained with Syto9 and propidium iodide confirmed an increase in the permeability of the bacterial membranes in the cultures incubated with the addition of carbazole derivative **2** at the concentration of 40 µg/mL compared with the untreated samples (Figure 7). In the control cultures of *S. aureus* and *P. aeruginosa*, only a small percentage of the cells were stained a red colour, which pointed to insignificant damage to the cell membranes. In the case of *S. aureus* and *P. aeruginosa* treated with the compound, about 35 and 91% of the cells were red, indicating that the membranes of these cells became more permeable to propidium iodide.

## 3. Discussion

Carbazole is a superior substrate for the synthesis of new antimicrobial compounds with more complex structures, which are particularly needed in the era of increasing drug resistance of microorganisms. For example, Gu et al. [6] synthesised carbazole derivatives of ursolic acid (Figure 1 **E**) as potential antitumor, antibacterial, and antifungal agents. The highest antimicrobial potential was exhibited by a derivative with a 5-fluoroindole and N-(dimethylamino)propyl amide chain, whose MIC values were in the range of 3.9–31.2 µg/mL [6]. Carbazole-oxadiazoles (Figure 1 **D**) also showed high antimicrobial potential, especially against the *S. aureus* strains. The growth of staphylococci was efficiently reduced by a 3,6-dibromocarbazole derivative, which was much more potent against the microorganisms than the tested antibiotic (norfloxacin). The MIC reached the values of 0.6–4.6 nmol/ml [5]. Among the N-substituted carbazole derivatives synthesised by Zhang et al. [10], 9-(4-(-imidazol-1-yl)butyl)-9*H*-carbazole showed the best antimicrobial activity, and its MIC values were 1–64 µg/mL. The substitution of carbazole with the thiazole ring and/or phenyl ring strongly increased its activity against different microorganisms [11]. Gu et al. [12] indicated that some derivatives of N-substituted 1*H*-dibenzo[a,c]carbazole demonstrate high antimicrobial activity, compared with the potential of antibiotics such as ketoconazole and amikacin. The antimicrobial properties of carvedilol, a cardiovascular drug containing a carbazole ring in the structure, had been described in our previous paper. Carvedilol (Figure 1 **F**) showed activity against Gram-positive strains, whereas the tested Gram-negative bacteria were resistant against its action [7]. An increase in ciprofloxacin activity was also observed after the simultaneous treatment of *S. aureus* cells with the antibiotic and carvedilol [13]. Carbazole derivative **2** showing structural similarity to carvedilol was synthesised in the presented studies. The compound was found to possess antibacterial activity comparable with that of carvedilol, although it was more active against the Gram-positive strains. Its MICs for *S. aureus* ATCC 6358, *S. epidermidis* ATCC 12228, *S. pyogenes* ATCC 19615 reached the values of 30, 50, and 40 µg/mL, respectively. In the case of carvedilol, the MIC values for the same strains were 40, 40, and 45 µg/mL. Thus, it was proved that the MBC values for carbazole derivative **2** were higher than those for carvedilol. Both compounds showed no significant activity against Gram-negative bacteria *E. coli* and *P. hauseri*, but they caused a significant reduction in the growth of the Gram-negative strain of *P. aeruginosa*. Growth inhibition in the cultures of *P. aeruginosa* supplemented with carbazole derivative **2** at the concentrations of 20 and 60 µg/mL was at the level of about 50 and 60%, respectively, whereas the same concentrations of carvedilol reduced *P. aeruginosa* growth by about 26 and 50%. The obtained results indicate that the weakening of the antibacterial effect of the compound against the Gram-positive strains and the enhancement of its action against *P. aeruginosa* are related to the structural differences between carbazole derivative **2** and carvedilol. The newly synthesised compound can be further modified to develop new derivatives effective in the treatment of *P. aeruginosa* infections, which is very important, as *P. aeruginosa* is a particularly dangerous microorganism causing acute inflammation of lungs and the infections of the skin and soft tissues. The presence of a wide variety of resistance mechanisms and the wide range of virulence factors make this strain a pathogen extremely difficult to control [14]. 

The LIVE/DEAD BacLight Bacterial Viability Kit was used in order to evaluate the mechanism of the antibacterial action of carbazole derivative **2**. The influence of the tested compound on the changes in the permeability of the bacterial membrane was examined based on dual staining with Syto9 and propidium iodide, which intercalate in the structure of nucleic acids. However, the dyes have a different ability to penetrate the cell membrane. Syto9 crosses the membrane of every bacterial cell, whereas propidium iodide can cross only disrupted membranes. These changes allow distinguishing between live and dead bacterial cells, which show green or red fluorescence, respectively [15]. The analysis of the obtained images suggests that the tested compound has a bacteriostatic effect against the Gram-positive strain of *S. aureus*. A significant reduction in *S. aureus* growth was detected in the cultures treated with carbazole derivative **2** at a concentration of 40 µg/mL, and about 35% of the visualised cells were dead. In the case of the Gram-negative *P. aeruginosa* strain, the growth inhibition was lower, but the cell mortality was higher, reaching 91%. Similar to carvedilol, which had been tested in our previous studies [7], the compound caused an increase in the permeability of the bacterial cell membrane. In the case of carvedilol (50 µg/mL), propidium iodide penetrated about 23% of *P. aeruginosa* cells, while in the cultures treated with carbazole derivative **2** at the concentration of 40 µg/mL, the membranes of about 91% of cells were damaged. Therefore, it seems that the structural differences between the tested compound and carvedilol are responsible for different mechanisms of their action against the Gram-positive *P. aeruginosa* strain. Although the inhibition of the growth of the mentioned bacteria was caused by both substances at a similar level, carbazole derivative **2** deteriorated the integrity of the bacterial membrane more strongly.

Haemolytic toxicity assay showed insignificant acute effects occurring in the red blood cells incubated with the addition of carbazole derivative **2**. These results correspond with the results obtained for antimicrobial carbazole–oxadiazoles [5]. A much more toxic effect was observed in the case of fibroblasts treated with the compound, where IC_50_ reached the value of 10 µg/mL. Xie et al. [5] showed low cytotoxic activity of carbazole–oxadiazoles in the Hek 293 T, LO2, and A549 cell lines. On the other hand, the carbazole derivatives of ursolic acid had IC_50_ values ranging from 0.62 to 100 µg/mL in hepatocarcinoma cell lines [6]. N-aryl carbazole derivatives substituted with an aminoguanidine or 1,4-dihydro-1,3,5-triazine moiety showed also differential cytotoxicity. The IC_50_ reached the values 0.4–42.1 µg/mL for human gastric cancer cells and human normal hepatic cells [4].

## 4. Materials and Methods

### 4.1. Materials

Mueller Hinton broth was obtained from Becton Dickinson (Warsaw, Poland). RPMI medium was purchased from Merck (Warsaw, Poland). DMEM and FBS were obtained from BioWest (Nuaillé, France). PBS and DMSO came from BioShop (Burlington, ON, Canada). The LIVE/DEAD Bacterial Viability Kit was obtained from Thermo Fisher Scientific (Warsaw, Poland).

### 4.2. Methods

#### 4.2.1. Synthesis of 4-(4-(Benzylamino)butoxy)-9H-carbazole **2**

To a solution of the 4-(4-bromobutoxy)-9*H*-carbazole **1** (0.050 g, 0.16 mmol) in dry acetonitrile (2.5 mL), benzylamine (0.045 mL, 0.41 mmol, 2.56 eq.), and KI (0.041 g, 0.25 mmol, 1.5 eq.) were added and the mixture was heated under reflux for 5 h. Afterwards, the suspension was filtered off, dichloromethane (5 mL) was added, and the mixture was washed with water (3 × 2 mL) and brine (1 × 2 mL). The organic phase was dried (MgSO_4_) and concentrated. The crude product was purified by chromatography on the silica gel columns with hexane–ethyl acetate mixture (1:1 to 1:2 *v/v)* to obtain pure carbazole **2** (0.045 g, 83%). 

IR (film, cm^−1^) ν_max_: 3409, 3287, 3082, 2939, 2868, 1626, 1606, 784, 751, 699; ^1^H NMR (600 MHz, CDCl_3_): δ = 8.34 (d, *J* = 7.7 Hz, 1H, H_arom_.), 8.12−8.09 (brs, 1H, NH), 7.44−7.39 (m, 2H, H_arom._), 7.38−7.33 (m, 5H, H_arom_.), 7.30−7.24 (m, 2H, H_arom._), 7.06 (d, *J* = 7.9 Hz, 1H, H_arom._), 6.67 (d, *J* = 7.9 Hz, 1H, H_arom._), 4.26 (t, *J* = 6.3 Hz, 2H, OC*H_2_*CH*_2_*), 3.86 (s, 2H, NHC*H_2_*Ph), 2.83 (t, *J* = 7.1 Hz, 2H, CH_2_C*H_2_*NH), 2.07 (t, *J* = 6.3 Hz, 2H, OCH_2_C*H_2_*CH_2_), 1.89 (t, *J* = 7.1 Hz, 2H, CH_2_C*H_2_*CH_2_NH), 1.80−1.65 (brs, 1H, N*H*CH_2_Ph); ^13^C NMR (151 MHz, CDCl_3_): δ = 155.53, 140.99, 138.76, 138.42, 128.60, 128.57, 127.44, 126.69, 124.91, 123.05, 122.73, 119.67, 112.67, 110.02, 103.48, 101.11, 67.59, 53.32, 48.52, 29.74, 27.22, 26.17. Anal. calcd. for C_23_H_24_N_2_O×0.5H_2_O: C, 78.16; H, 7.13; N, 7.92. Found: C, 78.43; H, 7.06; N, 7.70.

#### 4.2.2. Examination of Antimicrobial Activity

The antibacterial properties of carbazole derivative **2** were examined using the microdilution method against Gram-positive (*S. aureus* ATCC 29213, *S. aureus* ATCC 25923, *S. aureus* ATCC 6358, *S. aureus* ATCC 700699, *S. aureus* ATCC 43300, *S. epidermidis* ATCC 12228, *S. pyogenes* ATCC 19615) and Gram-negative (*E. coli* ATCC 25922, *P. hauseri* ATCC 13315, *P. aeruginosa* ATCC 15442) strains according to the Clinical and Laboratory Standards Institute (CLSI) standard M07 (11th Edition) for antimicrobial susceptibility testing of bacteria that grow aerobically. The growth of the tested bacteria untreated and treated with the compound was determined in Mueller–Hinton broth (MHB) in 96-well cell culture plates. Its antibacterial activity was tested in the concentration range of 10–70 µg/mL. The compound was dissolved in DMSO, and then the stock solution was diluted in an MHB medium. The inoculums of the tested strains with turbidity equal to 0.5 McFarland standard were diluted in MHB medium and added to the wells to achieve a final density of bacterial suspensions at 5 × 10^5^ CFU/ml. The turbidity of bacterial cultures was measured using a suspension turbidity meter (Densitometer DEN-1B, Biosan SIA, Latvia, Riga). The samples supplemented with the addition of carbazole derivative **2** and adequate abiotic and biotic control samples were incubated at 37 °C for 20 h in the dark. The antifungal properties of the compound were determined in RPMI 1640 medium according to CLSI standards M38 (3rd Edition) and M27 (4th Edition) for broth microdilution antifungal susceptibility testing of filamentous fungi and yeast, respectively. Its antifungal activity was tested on *A. flavus* ATCC 9643 and *C. albicans* ATCC 10231 in 96-well cell culture plates over the concentration ranges of 10–70 µg/mL. The stock solutions of the compound were prepared in DMSO and diluted in RPMI medium according to the CLSI guidelines. The spores of *A. flavus* were counted with a Thoma cell counting chamber and diluted in RPMI medium to achieve a final density of 2.5 × 10^4^ spores/ml. The inoculums of *C. albicans* with turbidity equal to 0.5 McFarland standard were diluted in RPMI medium and added to the wells to achieve a final density of yeast suspensions at 2.5 × 10^3^ CFU/ml. The turbidity of yeast cultures was measured using a suspension turbidity meter (Densitometer DEN-1B, Biosan SIA, Riga, Latvia). The samples supplemented with carbazole derivative **2** as well as abiotic and biotic controls were incubated at 37 °C for 48 h in the dark. After incubation, the growth of microorganisms untreated and treated with the compound was measured at *λ* = 630 nm using a microplate reader (Multiskan FC Microplate Photometer, ThermoFisher Scientific, Pudong, Shanghai, China). Microbial growth was calculated as a percentage of growth in the control samples based on the measured optical density of the cultures from three experiments (*n* = 3). Additionally, the minimum inhibitory concentration (MIC) and minimum bactericidal concentration/minimum fungicidal concentration (MBC/MFC) of carbazole derivative **2** were determined. The MIC was defined as the lowest concentration of the compound at which no growth of microorganisms was observed, whereas the MBC/MFC values were defined as the lowest concentrations that totally limited the viability of the microbial cells. The MIC and MBC/MFC values are expressed in µg/mL. 

#### 4.2.3. Assessment of the Haemolytic Activity of Carbazole Derivative **2**

Red blood cells (RBCs) were obtained from the Regional Centre of Blood Donation and Blood Treatment in Lodz (Poland). The haemolytic activity of carbazole derivative **2** was determined based on the spectrophotometric measurement of supernatants, which contained haemoglobin released from RBCs. RBCs were washed thrice with phosphate-buffered saline (PBS), and then the cells were suspended in PBS to obtain haematocrit 2.5%. Haemolytic properties were examined in carbazole derivative **2** concentrations 1–70 µg/mL. The controls including the cells suspended in PBS without the tested compound (negative control samples), and the cells suspended in pure deionised water (positive control samples) were also prepared. The samples and adequate controls were incubated in the dark at 37 °C for 1, 3, or 24 h. All test tubes were then centrifuged at 3000 rpm for 15 min and the absorbance of supernatants was measured spectrophotometrically at *λ* = 540 nm using a MultiskanTM FC Microplate Photometer (Thermo Fisher Scientific, Pudong, Shanghai, China). 

The haemolytic properties of carbazole derivative **2** were calculated using the following formula:

% Haemolysis= A_COMPOUND 2_/A_PC_ × 100%

where A_COMPOUND 2_ was the absorbance of samples incubated with the addition of carbazole derivative **2** and A_PC_ was the positive control absorbance.

The haemolytic potential was calculated as a percentage of haemolysis in the positive control samples (100%) from three experiments (*n* = 3).

#### 4.2.4. Determination of the Cytotoxic Activity of Carbazole Derivative **2**

The cytotoxic potential of carbazole derivative **2** on human cells was examined using the fibroblast BJ ATCC CRL-2522 cell line. The cells with a final density of 1 × 10^5^ cells/well were suspended in Dulbecco’s modified Eagle’s medium (DMEM) containing foetal bovine serum (10%) and antibiotics (penicillin 100 IU/mL and streptomycin 100 μg/mL). The final volume of the culture was 100 μL. The fibroblasts were incubated in 96-well microplates in a humidified atmosphere with 5% CO2 at 37 °C for 24 h. After incubation, the DMEM medium was replaced with a fresh medium with the addition of carbazole derivative **2** in the concentration range 1–70 μg/mL. Adequate control cultures untreated with the compound were also prepared. The plates were incubated again under the same conditions. After a 24-h incubation, the medium was removed from above the cells, and the wells were supplemented with MTT at a concentration of 500 μg/mL. Following a 2-h incubation under the same conditions, the solution was removed from the wells, and they were refilled with 100 μL of DMSO to dissolve the crystals of formazan. The viability of fibroblasts was calculated based on the spectrophotometric measurement of the cultures at *λ* = 550 nm using a SpectraMax i3x Multi-Mode Microplate Reader (Molecular Devices Ltd., Wokingham, UK). The results were presented as mean values of a percentage of fibroblast viability in control cultures untreated with carbazole derivative **2** with a standard deviation. The experiment with *n* = 3 was carried out in three independent repetitions.

#### 4.2.5. Confocal Analysis of Bacterial Cells Viability 

The viability of *S. aureus* ATCC 29213 and *P. aeruginosa* ATCC 15442 cells untreated and treated with carbazole derivative **2** at the concentration of 40 μg/ml was determined in the Laboratory of Microscopic Imaging and Specialised Biological Techniques (Faculty of Biology and Environmental Protection, University of Lodz, Lodz, Poland) using a Leica TCS SP8 microscope equipped with plan achromatic objectives (Leica Microsystems, Wetzlar, Germany) and with magnifications of 100× (Oil immersion). The bacterial cells incubated for 24 h without and with the addition of the tested compound were centrifuged at 10,000 rpm for 10 min and washed thrice with PBS. Then, the cells were stained using the dyes Syto9/Propidium Iodide according to the producer’s protocol. The fluorescence of PI and Syto9 was visualised at the Ex/Em maxima of 490/635 and 480/500 nm, respectively. 

#### 4.2.6. Statistical Analysis

The obtained results were expressed as the means with a standard deviation (SD). The one-way analyses of variance (ANOVA) with *p* < 0.05 were considered statistically significant calculations using Excel, Microsoft Office 365 Business Premium (Microsoft Corporation, Redmond, WA, USA).

## 5. Conclusions

The aim of our study was to investigate the toxicity and antimicrobial activity of a newly synthesised, functionalised carbazole derivative **2** (4-(4-(benzylamino)butoxy)-9*H*-carbazole). This new compound proved to be an effective antibacterial agent against Gram-positive bacterial strains. Almost complete inhibition of the microbial growth was observed in the cultures treated with the carbazole derivative at the concentration of 50 µg/mL. Another interesting finding is the activity of the tested compound against Gram-negative bacteria *P. aeruginosa*. In this case, the growth inhibition reached about 50% in the cultures supplemented with 20 µg/mL of the compound. Moreover, the cells stained with Syto9 and propidium iodide dyes showed over 90% mortality of *P. aeruginosa*. Therefore, the tested carbazole derivative **2** can be a promising structure for further modification and developing effective antimicrobial drugs. Future research should focus on assessing the biological activity of carbazole derivatives substituted with other types of amines and determining the relationship between their chemical structures and activity.

## Figures and Tables

**Table 1:** Antimicrobial activity of carbazole derivative **2** against bacteria and fungi. The minimum inhibitory concentration (MIC), minimum bactericidal concentration (MBC), and minimum fungicidal concentration (MFC) were expressed in µg/mL.

STRAIN	MIC	MBC/MFC
**Gram-positive bacteria**		
*S. aureus* ATCC 29213	30	>70
*S. aureus* ATCC 25923	40	>70
*S. aureus* ATCC 6358	30	>70
*S. aureus* ATCC 700699	40	70
*S. aureus* ATCC 43300	40	>70
*S. epidermidis* ATCC 12228	50	70
*S. pyogenes* ATCC 19615	40	60
**Gram-negative bacteria**		
*E. coli* ATCC 25922	>70	>70
*P. hauseri* ATCC 13315	>70	>70
*P. aeruginosa* ATCC 15442	>70	>70
**Fungi**		
*C. albicans* ATCC 10231	>70	>70
*A. flavus* ATCC 9643	>70	>70

## Data Availability

The data presented in this study are available on request from the corresponding author.
